# Computational prediction of plasma protein binding of cyclic peptides from small molecule experimental data using sparse modeling techniques

**DOI:** 10.1186/s12859-018-2529-z

**Published:** 2018-12-31

**Authors:** Takashi Tajimi, Naoki Wakui, Keisuke Yanagisawa, Yasushi Yoshikawa, Masahito Ohue, Yutaka Akiyama

**Affiliations:** 1 Department of Computer Science, School of Computing, Tokyo Institute of Technology, 2-12-1 W8-76 Ookayama, Meguro-ku, Tokyo, 152-8550 Japan; 2 Middle Molecule IT-based Drug Discovery Laboratory (MIDL), Tokyo Institute of Technology, RGBT2-A-1C 3-25-10 Tonomachi, Kawasaki-ku, Kawasaki city, Kanagawa 210-0821 Japan; 3 Molecular Profiling Research Center for Drug Discovery (molprof), National Institute of Advanced Industrial Science and Technology (AIST), 2-4-7 Aomi, Koto-ku, Tokyo, 135-0064 Japan

**Keywords:** Sparse modeling, Feature selection, Cyclic peptide, Biostability, Plasma protein binding (PPB)

## Abstract

**Background:**

Cyclic peptide-based drug discovery is attracting increasing interest owing to its potential to avoid target protein depletion. In drug discovery, it is important to maintain the biostability of a drug within the proper range. Plasma protein binding (PPB) is the most important index of biostability, and developing a computational method to predict PPB of drug candidate compounds contributes to the acceleration of drug discovery research. PPB prediction of small molecule drug compounds using machine learning has been conducted thus far; however, no study has investigated cyclic peptides because experimental information of cyclic peptides is scarce.

**Results:**

First, we adopted sparse modeling and small molecule information to construct a PPB prediction model for cyclic peptides. As cyclic peptide data are limited, applying multidimensional nonlinear models involves concerns regarding overfitting. However, models constructed by sparse modeling can avoid overfitting, offering high generalization performance and interpretability. More than 1000 PPB data of small molecules are available, and we used them to construct a prediction models with two enumeration methods: enumerating lasso solutions (ELS) and forward beam search (FBS). The accuracies of the prediction models constructed by ELS and FBS were equal to or better than those of conventional non-linear models (MAE = 0.167–0.174) on cross-validation of a small molecule compound dataset. Moreover, we showed that the prediction accuracies for cyclic peptides were close to those for small molecule compounds (MAE = 0.194–0.288). Such high accuracy could not be obtained by a simple method of learning from cyclic peptide data directly by lasso regression (MAE = 0.286–0.671) or ridge regression (MAE = 0.244–0.354).

**Conclusion:**

In this study, we proposed a machine learning techniques that uses low-dimensional sparse modeling to predict the PPB value of cyclic peptides computationally. The low-dimensional sparse model not only exhibits excellent generalization performance but also improves interpretation of the prediction model. This can provide common an noteworthy knowledge for future cyclic peptide drug discovery studies.

**Electronic supplementary material:**

The online version of this article (10.1186/s12859-018-2529-z) contains supplementary material, which is available to authorized users.

## Background

Cyclic peptides have attracted considerable interest from both the pharmaceutical industry and academia [1–4] for three main reasons. First, as with monoclonal antibody therapeutics, they can bind to target proteins with high affinities [5]. Second, they can interact with flat, shallow, and featureless surfaces of proteins or protein-protein interaction interfaces that are barely targeted by small molecule drugs [6]. Third, they have the potential for oral activity or oral bioavailability, similar to classical small molecule drugs [7–13]. More than 40 cyclic peptide drugs are currently approved for clinical use, and more than 20 cyclic peptides are in clinical development [14]. Most clinically approved cyclic peptides come from natural products, e.g., antibacterials or human peptide hormones [15–19]. Recently, de novo rational design techniques [20–22] and random screening techniques [23, 24] have facilitated development of novel cyclic peptide ligands for difficult targets [25–28].

Plasma protein binding (PPB), is the reversible binding of compounds to plasma proteins, and thus an equilibrium exists between bound and unbound forms. The fraction bound to plasma protein at equilibrium (*f*_*b*_) is an important pharmacokinetic property [29] since PPB is strongly related to the absorption, distribution, metabolism, excretion, and toxicity of such compounds. In most cases, only unbound portions of the compounds can be distributed into tissues, which then interact with the target proteins and are finally excreted from the blood [30, 31]. The candidate compounds that do not have appropriate PPB value are dropped in the later stages of drug discovery [32, 33]; however, experimental measurements are expensive and time-consuming. Moreover, the dropout of candidate compounds in the later stage increases the development costs. Therefore, it is necessary to estimate the PPB values of candidate compounds computationally in the early stages and prioritize development strategies.

As for small molecules, there are some reports related to the development of computational PPB prediction methods. PPB prediction methods are roughly classified into docking-based methods [34] and machine learning methods [35–38]. In docking-based methods, the PPB value is predicted using the molecular docking score on the basis of the pose in which compounds are docked to the plasma protein. Lexa et al. docked compounds to two major binding sites of human serum albumin (HSA) [34]. They reported that the weighted combination of the predicted LogP and docking score most accurately distinguishes between high-PPB-value compounds and low-PPB-value compounds, with an AUC of 0.94, when evaluated against a “strict set.” In machine learning methods, the model is trained on experimental PPB values of compounds, and the model predicts PPB values of unknown compounds. Previously, Ingle et al. used 1045 pharmaceutical data for model construction with support vector machines, k-nearest neighbors, and random forests and they evaluated these models against test data of 200 independent compounds and 406 environmentally relevant ToxCast chemicals [36]. They reported that the consensus model ensembled by these three non-linear models yielded mean absolute error (MAE) of 0.151–0.155 for pharmaceuticals and 0.110–0.131 for environmentally relevant chemicals. On the other hand, we found no reports on the development of PPB prediction methods for cyclic peptides. This implied the difficulty of predicting cyclic peptides due to paucity of experimental PPB data of cyclic peptides. It is also difficult to predict the binding poses of cyclic peptides owing to their large size and flexibility.

In general, it is considered that experimental PPB values of cyclic peptides is necessary to predict that of other cyclic peptides. However, as mentioned above, PPB data of cyclic peptides are insufficient, and it is very difficult to construct a prediction model with cyclic peptides. In practice, a prediction model trained on public cyclic peptide data is not very accurate, as discussed in the results section. Meanwhile, there are sufficient PPB data of small molecules. If small molecule data are informative for predicting cyclic peptides, machine learning method will work well. It is assumed that the physicochemical phenomena of PPB is same and important factors for explaining and governing PPB of both small molecules and cyclic peptides are universal. Feature selection, which is a task in machine learning, has the potential to extract such factors. Indeed, sparse modeling is a well-known method for feature selection. If these factors can be successfully extracted through feature selection, they could facilitate the construction of a model that predicts PPB values of cyclic peptides using PPB data of small molecules.

Here, we first propose the sparse model construction method to predict PPB values of cyclic peptides using small molecule data. The low-dimensional sparse model not only exhibits excellent generalization performance but also improves interpretation of the prediction model.

## Materials and methods

### Datasets

We used three types of datasets: small molecules, FDA-approved cyclic peptides, and cyclic peptides from in-house experiments. All molecules are available in the SMILES format with the fraction bound to plasma protein (*f*_*b*_) for PPB value listed in Additional file 1: Supplementary Table S1. *f*_*b*_ is a real number between 0 and 1. For some molecules, the *f*_*b*_ value is determined as not a specific value but a range [*f*_*b*min_, *f*_*b*max_]. We calculated the averaged value obtained by (*f*_*b*min_ + *f*_*b*max_)/2 and used it as *f*_*b*_ of the molecule.

The PPB values were converted into pseudo-equilibrium constant parameters (ln *K*_*a*_) for model construction, as there is a greater need for the resolution of higher *f*_*b*_ values (*f*_*b*_ > 0.8) than for that of moderate *f*_*b*_ values (*f*_*b*_ ≈ 0.5). The transformation equation is given by

$$ \ln {K}_a=C\ln \frac{f_b}{1-{f}_b}, $$

where *C* is a constant set to 0.3 as in a previous study [36]. The results of the ln *K*_*a*_ predictions were converted back to *f*_*b*_ for assessment of model accuracy according to a previous study [36]. To prevent divergence of the ln *K*_*a*_ value, *f*_*b*_ was scaled (*f*_*b*_ × 0.99 + 0.005) as in [37].

#### Small molecule dataset

We used pharmaceuticals with experimental *f*_*b*_ values originally corrected by Ingle et al. [36]. The training data and test data were split exactly as in [36]. We used 1017 out of 1045 training compounds and 194 out of 200 test compounds by removing compounds that could not calculate a part of molecular descriptors owing to failure of conformation generation. The former is the small molecule training data and the latter is the small molecule test data.

#### Public cyclic peptide drugs dataset

There are 24 cyclic peptides with PPB assay experimental results in DrugBank [39] (accessed November 6, 2017), which is a public database of FDA-approved drugs.

#### Original synthetic cyclic peptides dataset

As the number of publicly available data of cyclic peptide drugs is small compared to that of small molecule, we additionally designed and experimented with 16 cyclic peptides composed exclusively of natural amino acids. The synthetic cyclic peptide sequences are listed in Table 1. First, linear peptides were synthesized. Then, circularization was achieved by making a disulfide bond between N-terminal and C-terminal cysteine residues and confirmed by TOF/MS and HPLC analyses. Human PPB values *f*_*b*_ were determined by the equilibrium dialysis method [40]. Frozen human plasma was thawed immediately at room temperature. Then, the plasma was centrifuged at 3220 g for 10 min to remove clots and the supernatant was collected into a fresh tube. The working solutions of test compounds were prepared in DMSO at a concentration of 200 μM. Then, 3 μL of the working solution was removed for mixing with 597 μL of human plasma to achieve a final concentration of 1 μM (0.5% DMSO). The plasma samples were vortexed thoroughly. The dialysis membranes (HTD 96a/b Dialysis Membrane Strips MWCO 12-14 K, Cat. #1101, Batch# 1141 (12–17)) were soaked in ultrapure water for 60 min to separate the strips, then in 20% ethanol for 20 min, and finally in the dialysis buffer (100 mM sodium phosphate and 150 mM NaCl) for 20 min. The dialysis apparatus was assembled according to the manufacturer’s instructions. Each cell was filled with the spiked plasma sample and dialyzed against equal volume of the dialysis buffer. The assay was performed in duplicate. The dialysis plate was sealed and incubated in an incubator at 37 °C with 5% CO_2_ at 100 rpm for 6 h. At the end of incubation, the seal was removed and 50 μL of samples from both buffer and plasma chambers were transferred to wells of a 96-well plate. 50 μL of blank plasma was added to each buffer sample and an equal volume of phosphate buffered saline was supplemented to the collected plasma sample. 300 μL of room temperature quench solution (acetonitrile containing internal standards (IS, 100 nM Alprazolam, 500 nM Labetalol and 2 μM Ketoprofen)) was added to precipitate protein. Samples in the plate were vortexed for 5 min and centrifuged at 3220 g for 30 min at 4 °C. Then, the supernatant was transferred to a new 96-well plate with 100 μL or 200 μL water (depending on the LC-MS signal response and peak shape) for LC-MS/MS analysis.

**Table 1:** Sequences of synthetic cyclic peptides. Circularization was achieved by making a disulfide bond between N-terminal and C-terminal cysteine residues. These peptides are available in the SMILES format listed in Additional file 1: Table S1

Peptides	Sequences									Exp. PPB (*f*_b_^*^)
Pep.1	Cys	Tyr	Phe	Gln	Asn	Pro	Arg	Gly	Cys	0.242
Pep.2	Cys	Tyr	Ile	Gln	Asn	Pro	Leu	Gly	Cys	0.005
Pep.3	Cys	Ala	Trp	Lys	Val	Thr	Cys			0.00040
Pep.4	Cys	Phe	Pro	Phe	Trp	Lys	Tyr	Cys		0.616
Pep.5	Cys	Trp	Arg	Pro	Arg	Val	Ala	Arg	Cys	0
Pep.6	Cys	Phe	Phe	Trp	Lys	Thr	Thr	Cys		0.263
Pep.7	Cys	Lys	Leu	Leu	Lys	Lys	Thr	Cys		0
Pep.8	Cys	Tyr	Tyr	Tyr	Tyr	Tyr	Tyr	Tyr	Cys	0.855
Pep.9	Cys	Ala	Gly	Leu	Val	Leu	Ala	Ala	Cys	0
Pep.10	Cys	Trp	Val	His	Pro	Gln	Phe	Glu	Cys	0.367
Pep.11	Cys	Asn	Gln	Pro	Trp	Gln	Cys			0
Pep.12	Cys	Ser	Phe	Asp	Asp	Trp	Leu	Ala	Cys	0.800
Pep.13	Cys	Tyr	Leu	Ala	Glu	Tyr	His	Gly	Cys	0.349
Pep.14	Cys	Ala	Pro	Ala	Trp	Ala	His	Gly	Cys	0.074
Pep.15	Cys	Phe	Val	Tyr	Ser	Ala	Val	Cys		0.153
Pep.16	Cys	Arg	Ile	Lys	Arg	Tyr	Cys			0.151

### Molecular descriptors

To characterize molecules, we calculated 2D descriptors of the compounds and 3D descriptors of conformers of the compounds. The descriptors were calculated using molecular_descriptors.py and QikProp provided by Schrödinger, LLC [41]; there are 281 descriptors in total. As conformers are required to generate 3D descriptors of compounds, the most stable conformation of each compound was generated from SMILES by LigPrep (Schrödinger, LLC) [42]. The descriptors consist of physical properties (e.g., LogS, LogP, and ASA) and topological descriptors based on the graphical representation of the molecules. All the descriptors were standardized to mean *μ* = 0 and variance *σ*^2^ = 1 with reference to small molecule training data.

### Enumeration of extracted descriptor sets

**Fig. 1:**
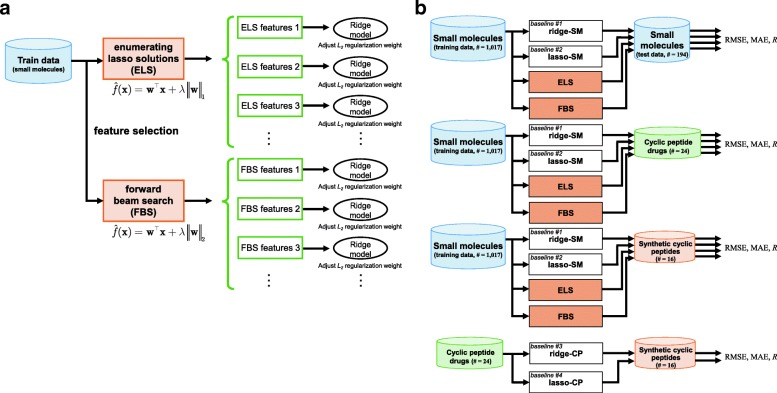
Outline of the construction of the prediction models for PPB in this study. **a** The flow of feature selection and model construction using enumerating lasso solutions (ELS) and forward beam search (FBS). **b** The flow of evaluation for prediction performance on baseline models and proposed models

It is important to extract better descriptors in terms of robustness and interpretation of the prediction model. The biophysical basis of PPBs must be the same for small molecules and cyclic peptides. However, in this case, a result of feature selection strongly depends on the training data (small molecules). In other words, descriptors specific to small molecules (i.e., those that cannot represent cyclic peptides) will be chosen. Thus, we present multiple results of feature selection. Enumerating lasso solutions (ELS) and forward beam search (FBS) were used as feature selection methods, and the generated models were compared to baseline models trained on all descriptors. Feature selection was performed using the small molecule dataset, followed by application of the extracted subsets of descriptors to two cyclic peptide datasets. This process is needed because there are insufficient cyclic peptide data for extracting and verifying the descriptors. An outline of the feature selection and sparse modeling is shown in Fig. 1(a).

#### Enumerating lasso solutions

Hara and Maehara [43] proposed a sparse modeling algorithm for enumerating solutions to the lasso regression problem (least-squares method with *L*_1_ regularization). This enumerating lasso solutions (ELS) algorithm is summarized in Algorithm 1, where *n* is the number of dimensions of a feature space, *P* = {1, 2, …, *n*} is a set representing indices of all the features, *Lasso*(*S*) is the function that calculates the lasso solution that allows coefficients of features included in *S* ⊆ *P* to be non-zero, and *supp*(*β*) is a set of features with non-zero coefficients in the enumerated lasso solution *β*. This algorithm calculates a lasso solution different from *β* by removing the features of *supp*(*β*) one by one from *S.* The output candidate is added as a tuple (*β*, *S*, *F*) to the priority queue for the objective function value of the lasso. *F* is a set of features removed from *P*. The weight parameter of the *L*_1_ regularization term is related to the sparseness of the lasso solution. The algorithm can output multiple results of feature selection, whereas ordinary lasso regression outputs only a single result.

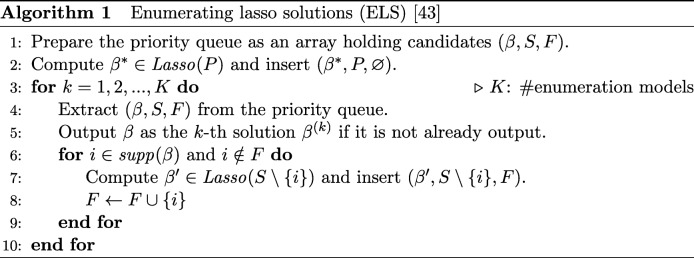



#### Forward beam search

We also used forward beam search (FBS), which can output multiple results by applying beam search to the forward-stepwise extraction. A ridge regression model (least-squares method with *L*_2_ regularization) was used for each descriptor set. The residual sum of squares was used for the loss of each model. When the search was completed, the best results of the descriptor sets were output in ascending order of loss. This FBS algorithm is summarized in Algorithm 2. *P* = {1, 2, …, *n*} is a set representing indices of all the features. *Ridge*(*S*) is the function that calculates the ridge solution.

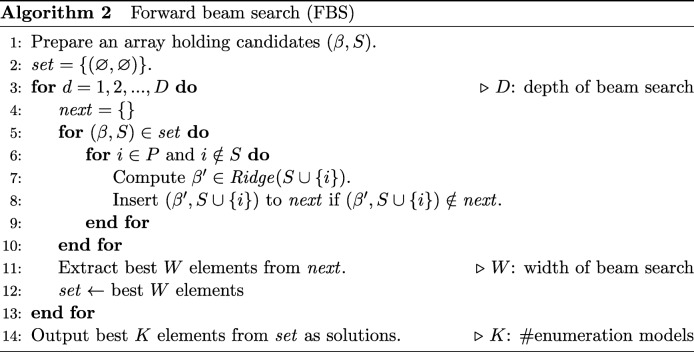



#### Parameters of ELS and FBS

To balance the prediction accuracy and interpretability, we selected the number of descriptors in an extracted set to be around 5. For ELS, the weight parameter of the *L*_1_ regularization term was set to 0.13 so that the number of features with non-zero coefficients in lasso’s global optimal solution was 5. The search depth of FBS was set to *D* = 5 so that each extracted set has just 5 descriptors. In addition, the weight parameter of the *L*_2_ regularization term in FBS was set to 1.0. Furthermore, the number of enumerations of each method was set to *K* = 200, and the beam width of FBS was set to *W* = 300, which are determined for the following reasons. In the general tendency, a larger value of *K* increases the possibility of finding a better model. The number of descriptors we selected is around 4 to 5, and *K* needs to be set between 100 and 300. In this local range, selected features were the same. In other words, the prediction accuracy was the same; thus, *K* was set to 200. Similarly, a larger value of *W* will also result in a better model because the search range becomes wider. However, in the range of *W* = 200–400, the prediction accuracy was the same; thus, *W* was set to 300.

### Model evaluation

The models were evaluated and compared based on the root mean squared error (RMSE) of the predicted *f*_*b*_, the mean absolute error (MAE) of the predicted *f*_*b*_, and the correlation coefficient (*R*) of ln *K*_*a*_. These metrics are defined in the equations below:

$$ \mathrm{RMSE}=\sqrt{\frac{\sum_i^N{\left({f}_{b,i}-{f}_{b,i}^{\ast}\right)}^2}{N}}, $$

$$ \mathrm{MAE}=\frac{\sum_i^N\left|{f}_{b,i}-{f}_{b,i}^{\ast}\right|}{N}, $$

$$ R=\frac{\sum_i^N\left\{{\left(\ln {K}_a\right)}_i-\overline{\ln {K}_a}\right\}\left\{{\left(\ln {K}_a\right)}_i^{\ast }-\overline{\ln {K_a}^{\ast }}\right\}}{\sqrt{\sum_i^N{\left\{{\left(\ln {K}_a\right)}_i-\overline{\ln {K}_a}\right\}}^2{\sum}_i^N{\left\{{\left(\ln {K}_a\right)}_i^{\ast }-\overline{\ln {K_a}^{\ast }}\right\}}^2}}, $$

where *N* is the number of data, ${f}_{b,i}$ is the predicted value of *f*_*b*_ in compound *i*, ${f}_{b,i}^{\ast }$ is the experimental value of *f*_*b*_ in compound *i*, (ln *K*_*a*_)_*i*_ is the ln *K*_*a*_ value converted from *f*_*b*,*i*_, ${\left(\ln {K}_a\right)}_i^{\ast }$ is the ln *K*_*a*_ value converted from ${f}_{b,i}^{\ast }$, and $\overline{\ln {K}_a}$ and $\overline{\ln {K_a}^{\ast }}$ are the mean values of (ln *K*_*a*_)_*i*_ and ${\left(\ln {K}_a\right)}_i^{\ast }$, respectively.

### Model construction

#### Baseline models

To verify the effectiveness of the proposed method, four baseline models were constructed (white boxes in Fig. 1(b)). These models were trained on two different types of datasets (small molecule training dataset or cyclic peptide drug dataset), and baseline performances were obtained by predicting three datasets: small molecule test dataset (SM), cyclic peptide drug dataset (CP), and synthetic cyclic peptide dataset (SCP). Detailed schemes are described in Fig. 1(b). The ridge model with all features and lasso model with five features were used to construct baseline models. Hereafter, we refer to their baseline models “regressor name-training data” (e.g., ridge-SM). In baseline #1 (ridge-SM) and baseline #2 (lasso-SM), the models were trained on the small molecule training dataset, and predicted three datasets. In baseline #3 (ridge-CP) and baseline #4 (lasso-CP), PPB values of the cyclic peptide drug dataset and the synthetic cyclic peptide dataset were predicted. When predicting cyclic peptide drug dataset by ridge-CP and lasso-CP, leave-one-out cross-validation (LOOCV) was also conducted.

#### Proposed models

To compare the two feature selection methods, ridge regression models were generated for all extraction results obtained using the small molecule training data. The *L*_2_ regularization weight parameter was adjusted on the basis of 3-fold cross-validation with small molecule training data for each result of the descriptor subsets. This cross-validation is only used to select model parameters during training. The models having the smallest RMSE of test data from each of the two methods of feature selection were selected as the proposed models because it was assumed that the model of best prediction of unknown data explains the PPB. Under the same conditions as those for ridge-SM and lasso-SM, the prediction accuracies of the proposed models were also evaluated.

## Results

### Distribution of the datasets

**Fig. 2:**
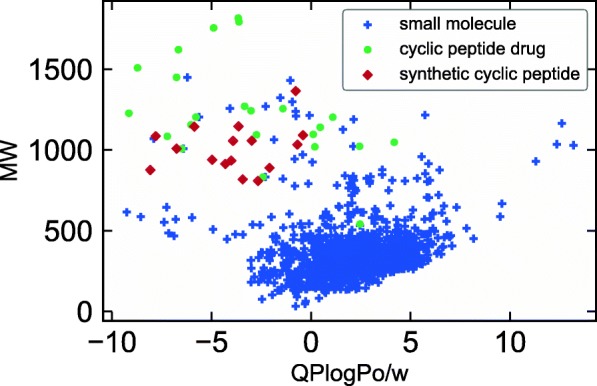
Scatter plot for three datasets, namely small molecules, cyclic peptide drugs, and synthetic cyclic peptides, in molecular weight (MW) and octanol/water partition coefficient (QPLogPo/w) space

To visualize the datasets, a scatter plot with molecular weight (MW) and octanol/water partition coefficient (QPLogPo/w) is shown in Fig. 2. It was found that most of the cyclic peptides are larger than the small molecules. The distribution of the synthetic cyclic peptides was slightly similar to that of the small molecule dataset, compared to that of the cyclic peptide drug dataset.

### Small molecules PPB modeling

**Fig. 3:**
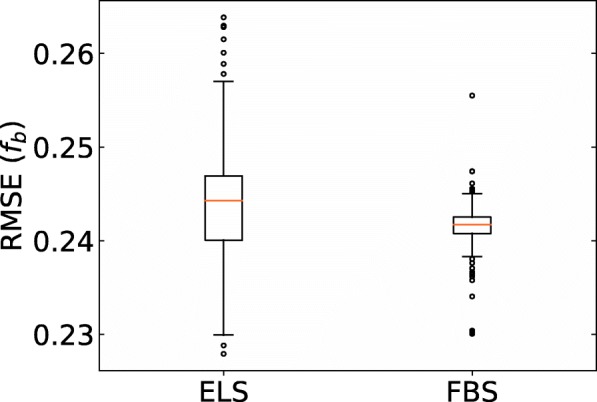
Boxplots of RMSE of each model for prediction of small molecule test data from small molecule training data. ELS stands for enumerating lasso solutions, and FBS stands for forward beam search. Each box plot indicates the median value (orange line), first and last quartile, and the outliers (circles). Here, *IQR* denotes the interquartile range. The ends of the whiskers represent the lowest datum still within 1.5 × *IQR* of the lower quartile, and the highest datum still within 1.5 × *IQR* of the upper quartile

**Fig. 4:**
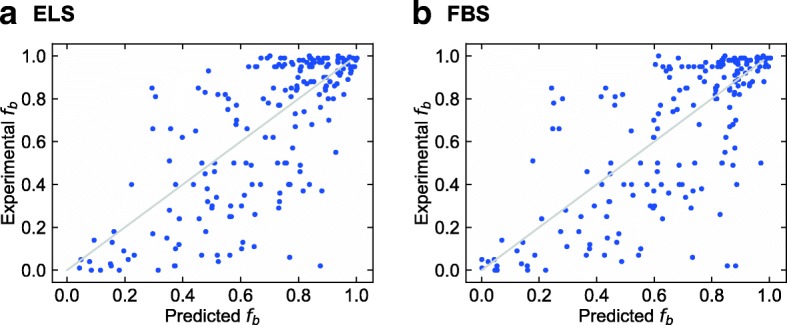
Scatter plots of the experimental values of small molecule test data versus predicted values of that for models constructed by ELS and FBS. The models were trained on small molecule training data

Hereafter, we present the prediction results in each situation described in Fig. 1(b). The results of predicting the *f*_*b*_ values of small molecule dataset are listed in Table 2, and this situation is the same as that in the work of Ingle et al. The prediction accuracy of ridge-SM and lasso-SM is similar to that in the work of Ingle et al. As for the proposed ELS and FBS, it was found that the prediction accuracy is as good as that of ridge-SM and lasso-SM. Figure 3 shows the boxplots of each RMSE in *f*_*b*_ of the small molecule test data with 200 different models. Although the prediction accuracy of both ELS and FBS varied according to the selected descriptors, the variation in the RMSE of ELS and FBS was around 0.02 and 0.01, respectively. Figure 4 shows a scatter plot of the PPB prediction results for ELS and FBS. The correlation coefficients between experimental *f*_*b*_ and estimated *f*_*b*_ in ELS and FBS were 0.714 and 0.725, respectively.

**Table 2:** PPB prediction results of small molecules for sparse modeling by ELS and FBS compared to the baseline results. These situations are shown in a part of Fig. 1(b)

Method	Training set	Test set	RMSE (*f*_*b*_)	MAE (*f*_*b*_)	*R* (ln *K*_*a*_)
ridge-SM (baseline #1)	Small molecules (training data)	Small molecules (test data)	0.212	0.155	0.781
lasso-SM (baseline #2)	Small molecules (training data)	Small molecules (test data)	0.233	0.176	0.707
ELS	Small molecules (training data)	Small molecules (test data)	0.228	0.172	0.714
FBS	Small molecules (training data)	Small molecules (test data)	0.230	0.167	0.725
Non-linear model [36]	Small molecules (training data)	Small molecules (test data)	0.225–0.251	0.155–0.177	0.707–0.787

### Simple ridge and lasso regression for cyclic peptides PPB directly

The prediction results for ridge-CP and lasso-CP are listed in Table 3. The prediction accuracy degraded compared to that of ridge-SM and lasso-SM, as the number of the data is much smaller than that of the small molecule dataset. Internal validation with the cyclic peptide drug dataset was carried out in LOOCV (ridge-CP-LOO and lasso-CP-LOO in Table 3) and the results were allowable (MAE = 0.244–0.286). For the prediction model constructed based on the cyclic peptide drug dataset, however, the prediction model could not predict for the synthetic cyclic peptide dataset at all. In this case, the MAE of ridge-CP and lasso-CP was 0.354 and 0.627, respectively. Originally, the number of cyclic peptide samples was extremely small. This implies that using cyclic peptides as training data is inappropriate unless the number of data is increased.

**Table 3:** Prediction results for ordinary lasso sparse modeling and ridge regression as baseline results under the several situations shown in a part of Fig. 1(b)

Method	Training set	Test set	RMSE (*f*_*b*_)	MAE (*f*_*b*_)	*R* (ln *K*_*a*_)
ridge-CP-LOO (baseline #3)	Cyclic peptide drugs (LOOCV)	Cyclic peptide drugs (LOOCV)	0.338	0.244	0.418
lasso-CP-LOO (baseline #4)	Cyclic peptide drugs (LOOCV)	Cyclic peptide drugs (LOOCV)	0.358	0.286	0.289
ridge-CP (baseline #3)	Cyclic peptide drugs (# = 24)	Synthetic cyclic peptides (# = 16)	0.413	0.354	0.442
lasso-CP (baseline #4)	Cyclic peptide drugs (# = 24)	Synthetic cyclic peptides (# = 16)	0.688	0.627	0.069

### Prediction for cyclic peptide drugs with small molecules using sparse modeling

**Fig. 5:**
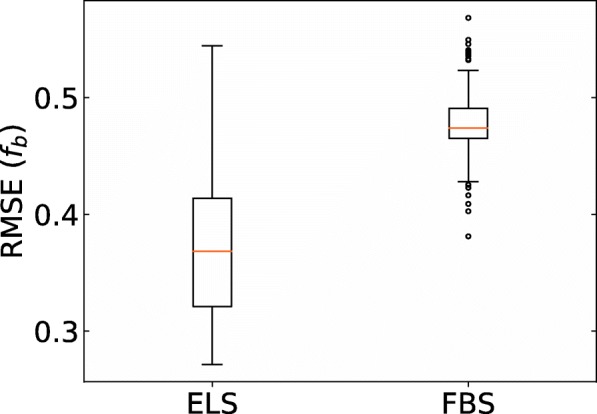
Boxplots of RMSE of each model for prediction of cyclic peptide drugs. Each box plot indicates the median value (orange line), first and last quartile, and the outliers (circles). Here, *IQR* denotes the interquartile range. The ends of the whiskers represent the lowest datum still within 1.5 × *IQR* of the lower quartile, and the highest datum still within 1.5 × *IQR* of the upper quartile

The results of predicting PPB values of cyclic peptide drugs using the models constructed with the small molecule training data are listed in Table 4. In particular, ridge regression prediction ridge-SM failed (MAE = 0.442), indicating the effectiveness of sparse modeling. Among the constructed models, ELS predicted PPB values of cyclic peptide drugs more accurately than other methods (MAE = 0.216). The random prediction by output *f*_*b*_ from uniform distribution was compared with ELS and FBS. The cyclic peptide dataset is predicted 10,000 times through random prediction. The average MAE is 0.374. Compared with this value, ELS (MAE = 0.216, one-sided *P*-value = 0.0013 with unpaired *t*-test) and FBS (MAE = 0.288, one-sided *P*-value = 0.044 with unpaired *t*-test) are significantly better. Figure 5 shows a boxplot of RMSE of *f*_*b*_ when predicting ln *K*_*a*_ of cyclic peptide drug data in all models. Although the RMSEs of the worst model in ELS and FBS were similar, the RMSE of the best model in ELS was less than that of FBS.

**Table 4:** PPB prediction results of cyclic peptide drugs for sparse modeling by ELS and FBS compared to the baseline results. These situations are shown in a part of Fig. 1(b). The values with asterisk represent the best prediction performance in each evaluation criterion, and ridge-CP-LOO and lasso-CP-LOO lines are reproduced from Table 3

Method	Training set	Test set	RMSE (*f*_*b*_)	MAE (*f*_*b*_)	*R* (ln *K*_*a*_)
ridge-SM (baseline #1)	Small molecules (training data)	Cyclic peptide drugs (# = 24)	0.528	0.442	0.120
lasso-SM (baseline #2)	Small molecules (training data)	Cyclic peptide drugs (# = 24)	0.321	0.251	0.444
ELS	Small molecules (training data)	Cyclic peptide drugs (# = 24)	0.272*	0.216*	0.464*
FBS	Small molecules (training data)	Cyclic peptide drugs (# = 24)	0.381	0.288	0.270
ridge-CP-LOO (baseline #3)	Cyclic peptide drugs (LOOCV)	Cyclic peptide drugs (LOOCV)	0.338	0.244	0.418
lasso-CP-LOO (baseline #4)	Cyclic peptide drugs (LOOCV)	Cyclic peptide drugs (LOOCV)	0.358	0.286	0.289

### Prediction for synthetic cyclic peptides with small molecules using sparse modeling

**Fig. 6:**
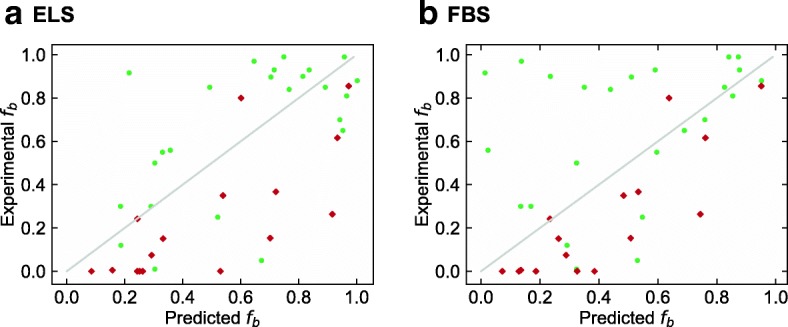
Scatter plots of the experimental values of cyclic peptide data versus predicted values of that for models constructed by ELS and FBS. The models were trained on small molecule training data. The green dots denote cyclic peptide drugs and the red rectangles denote synthetic cyclic peptides

The results of predicting the synthetic cyclic peptide dataset using the models trained on the small molecule training data are listed in Table 5. As already seen in Fig. 2, the spatial distribution of the synthetic cyclic peptide dataset overlaps with that of the small molecule dataset; thus, this prediction result also shows good accuracy. Ridge-SM, lasso-SM, and ELS showed nearly equivalent accuracy, and FBS was particularly accurate (MAE = 0.194). The prediction accuracies of ridge-CP and lasso-CP were worse. Therefore it is reasonable to use a small molecule dataset with abundant data for predicting cyclic peptide PPB. Figure 6 shows a scatter plot of predicted *f*_*b*_ and experimental *f*_*b*_ of the two cyclic peptide datasets in each model with the smallest RMSE.

**Table 5:** PPB prediction results of synthetic cyclic peptides for sparse modeling by ELS and FBS compared to the baseline results. These situations are shown in a part of Fig. 1(b). The values with asterisk represent the best prediction performance in each evaluation criterion, and ridge-CP and lasso-CP lines are reproduced from Table 3

Method	Training set	Test set	RMSE (*f*_*b*_)	MAE (*f*_*b*_)	*R* (ln *K*_*a*_)
ridge-SM (baseline #1)	Small molecules (training data)	Synthetic cyclic peptides (# = 16)	0.321	0.263	0.761
lasso-SM (baseline #2)	Small molecules (training data)	Synthetic cyclic peptides (# = 16)	0.276	0.228	0.714
ELS	Small molecules (training data)	Synthetic cyclic peptides (# = 16)	0.319	0.269	0.748
FBS	Small molecules (training data)	Synthetic cyclic peptides (# = 16)	0.230*	0.194*	0.805*
ridge-CP (baseline #3)	Cyclic peptide drugs (# = 24)	Synthetic cyclic peptides (# = 16)	0.413	0.354	0.442
lasso-CP (baseline #4)	Cyclic peptide drugs (# = 24)	Synthetic cyclic peptides (# = 16)	0.688	0.627	0.069

## Discussion

### Comparison of ELS and FBS

**Fig. 7:**
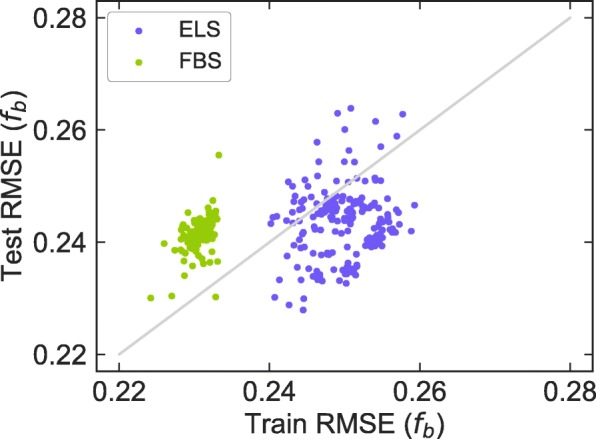
Scatter plot of RMSE values of small molecule training data versus small molecule test data for models constructed by ELS and FBS

Interestingly, the best model of ELS outperformed that of FBS in terms of prediction of cyclic peptides (Table 4), unlike the results of prediction with small molecules (Table 2) and the original synthetic cyclic peptides (Table 5). We analyzed the prediction accuracies in detail. The relationship between the prediction error of small molecule training data and that of small molecule test data is shown in Fig. 7. According to the figure, the averaged prediction accuracy for small molecule test data is nearly the same as that for training data in ELS, whereas the averaged prediction accuracy for small molecule test data is worse than that for the training data in FBS. This means that the models based on FBS are more overfitted to the training data. Thus, we concluded that models of ELS are more robust in predicting diverse molecules such as cyclic peptides, which have properties different from those of training data (small molecules). ELS can generate models having high generalization ability. This is important when predicting for cyclic peptides with the model trained on small molecules.

Interestingly, the prediction tendencies of the best models of ELS and FBS are different. To compare the bias of prediction in cyclic peptide drug dataset and synthetic cyclic peptide dataset (Fig. 6(a) and 6(b)), $\Pr \left({f}_b-{f}_b^{\ast }>0.3\right)$ and $\Pr \left({f}_b^{\ast }-{f}_b>0.3\right)$ are calculated by counting samples, where *f*_*b*_ represents the predicted value and ${f}_b^{\ast }$ represents the experimental value. In ELS, $\Pr \left({f}_b-{f}_b^{\ast }>0.3\right)=3/40=0.075$ and $\Pr \left({f}_b^{\ast }-{f}_b>0.3\right)=7/40=0.175$. In FBS, $\Pr \left({f}_b-{f}_b^{\ast }>0.3\right)=8/40=0.200$ and $\Pr \left({f}_b^{\ast }-{f}_b>0.3\right)=6/40=0.150$. These values seem to indicate that ELS often predicts lower *f*_*b*_ than experimental *f*_*b*_ but FBS exhibits an opposite tendency to that of ELS.

### Feature set and prediction accuracy of the most predictable model

The extracted descriptors by our best models for small molecule test data of the two feature selection methods are summarized in Tables 6 and 7. The physical descriptors were compared with those extracted in the previous study by Ingle et al. [36]. Interestingly, most of the physical descriptors, such as the charge descriptor (PEOE), the surface descriptor (SASA, PISA), and the partition coefficient (LogPo/w, QPLogPo/w), are consistent; hence, we confirmed that feature selection methods worked well and that these descriptors may be important for predicting PPB values.

**Table 6:** Descriptors and regression coefficients of the best model for cyclic peptide drugs obtained by ELS trained on small molecule training data

Descriptor	Category	Regression coef.
Radial centric	Topological descriptors	0.06
PEOE6	Physical property	0.10
PISA	Physical property	0.11
QPLogPo/w	Physical property	0.25

**Table 7:** Descriptors and regression coefficients of the best model for cyclic peptide drugs obtained by FBS trained on small molecule training data

Descriptor	Category	Regression coef.
PEOE8	Physical property	−0.42
PEOE9	Physical property	−0.39
Xu	Topological descriptors	0.75
Percent Human Oral Absorption	Physical property	0.18
QPLogPo/w	Physical property	0.36

### PCA analysis with extracted descriptors

**Fig. 8:**
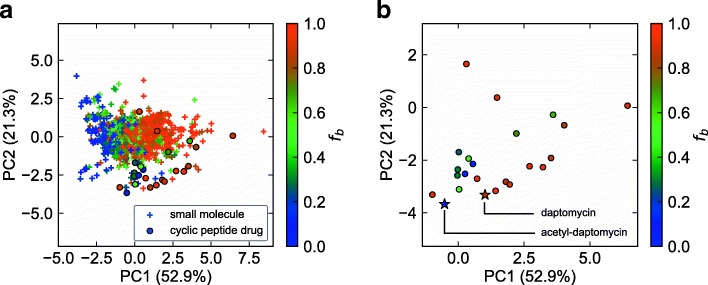
PCA results with descriptors that are in the best model for prediction of cyclic peptide drugs obtained from ELS trained on small molecule training data. **a** Cyclic peptide drugs and small molecules are shown and (**b**) only cyclic peptide drugs are shown. Daptomycin and acetyl-daptomycin are indicated with stars. The proportion of total variation explained by each principal component is indicated by the percentage in parenthesis

The distances between the molecules in the linear models can be estimated with the distance of the scatter plot of principal component analysis (PCA). Thus, we applied PCA for all molecules with ELS extracted descriptors that performed the best for cyclic peptide drug data. Figure 8 shows scatter plots of first and second principal components (PC1, PC2) of ELS extracted descriptors. Figure 8(a) shows small molecules and cyclic peptide drugs in the same PC space. Figure 8(b) shows only cyclic peptide drugs for the readers’ benefit. Both small molecules and cyclic peptide drugs tend to have low *f*_*b*_ when PC1 and PC2 are smaller and high *f*_*b*_ when PC1 and PC2 are larger. Therefore, these features are considered to be similar explanations for PPB in both small molecule compounds and cyclic peptides. Although the region in which cyclic peptides are plotted is biased compared to small molecule compounds, this feature set may partially represent the important structure of cyclic peptides.

### Good and bad cases for prediction

**Fig. 9:**
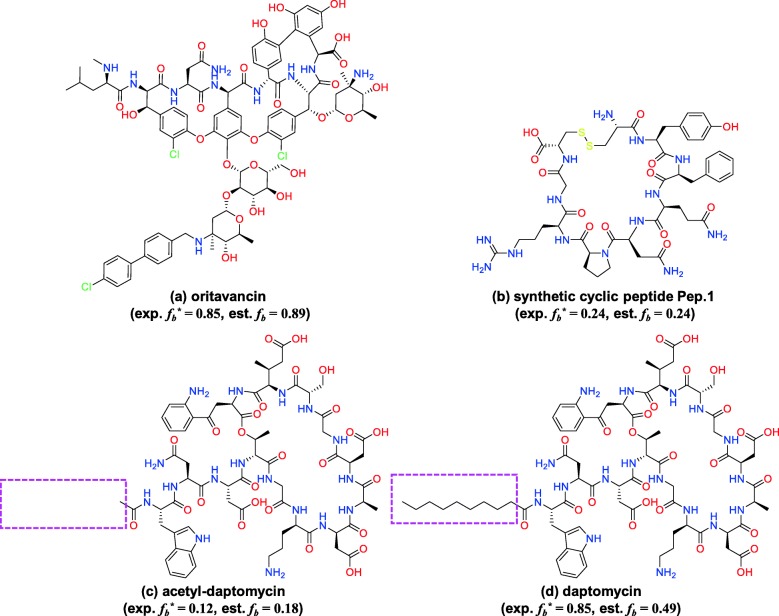
Structures of **a** oritavancin (cyclic peptide drug), **b** Pep.1 (synthetic cyclic peptide), **c** acetyl-daptomycin (cyclic peptide drug), and **d** daptomycin (cyclic peptide drug), with the fraction of PPB (*f*_*b*_). The dashed magenta rectangles highlight the structural difference of **c** and **d**

Figure 9 shows four cyclic peptides as good and bad prediction cases. Oritavancin (Fig. 9(a)) is typical good case (experimental *f*_*b*_ is 0.85 and estimated *f*_*b*_ is obtained as 0.84 by ELS). Pep.1 of the synthetic cyclic peptides (Fig. 9(b)) is also a good case (experimental *f*_*b*_ is 0.24 and estimated *f*_*b*_ is obtained as 0.18 by ELS). Acetyl-daptomycin succeeded in predicting PPBs, as shown in Fig. 9(c). Daptomycin, shown in Fig. 9(d), is an example of a bad case and it is quite suggestive. Although the only difference between acetyl-daptomycin and daptomycin is the absence or presence of the alkyl chain (Figs. 9(c) and (d), dashed magenta rectangle area), the PPB values of these two cyclic peptides are totally different. The PPB value of daptomycin is 0.85, whereas that of acetyl-daptomycin is 0.12. A previous study related to these two cyclic peptides reported that the alkyl chain of daptomycin forms a hydrophobic interaction with HSA [44]. Therefore the absence or presence of the alkyl chain is important. The PCA plot based on the selected descriptors showed that the distance between the points corresponding to daptomycin and acetyl-daptomycin was not large (Fig. 8(b)). Thus, it can be assumed that extracted descriptors do not evaluate the effect of the alkyl chain sufficiently well.

We need some new descriptors that express a local or partial structure to predict the PPB value of cyclic peptides. Toward this end, it could be assumed that taking particular note of residues that are most likely to bind to HSA or other plasma proteins is a reasonable approach. We need to define “residues that are most likely to bind” and calculate descriptors covering these residues and surrounding structures. The difference in local alkyl chain was important for PPB in the case of Fig. 9. Xu index appeared in FBS model (Table 7) is known as one of topological descriptors for the molecular backbone [45, 46]. Therefore, it may be possible to extract the difference well by evaluating it for the local structure. The correlation coefficient between *f*_*b*_ and Xu index is 0.452. This medium correlation perhaps show that Xu index is important for providing an explanation of PPB. However, the correlation between molecular weight and Xu index is very high (coefficient = 0.990). It is important to propose a novel descriptor that not merely reflects the total weight like Xu index but can express local structural difference. Accordingly, it might be possible to generate descriptors that focus on the local or partial structure of cyclic peptides and stand for the binding between plasma proteins and cyclic peptides.

## Conclusions

This study aimed to predict the fraction bound to plasma proteins of cyclic peptides by using sparse modeling techniques in machine learning. Enumeration methods were utilized to predict PPB values of cyclic peptides with the model trained on experimental PPB data of small molecules. Two enumeration methods, enumerating lasso solutions (ELS) and forward beam search (FBS), were compared to four baseline models constructed using ridge and ordinal lasso regressions. Their prediction accuracies were evaluated with two cyclic peptide datasets: public cyclic peptide drugs obtained from DrugBank database and original cyclic peptides synthesized in this study, and proposed models showed better performance than baseline models (ELS obtained MAE value of 0.216 and 0.269 in cyclic peptide drugs and synthetic cyclic peptides, respectively, FBS obtained MAE value of 0.288 and 0.194 in cyclic peptide drugs and synthetic cyclic peptides, respectively). The prediction model constructed by the sparse modeling techniques, ELS and FBS, well achieved the aim of this study; that is, predicting PPB value of cyclic peptides.

The directions for future work are as follows. It is known that the local structure is important for predicting PPB of cyclic peptides, but the descriptor set of the most accurate model can only partly represent this structure. Thus, we shall investigate an example of a method for constructing the features that can represent the local structure better. In addition, gathering experimental PPB values of cyclic peptides is important for further discussion and for improving the accuracy of the prediction model.

## Additional file

Additional file 1: **Table S1.** referred to in the main article. http://www.bi.cs.titech.ac.jp/giw2018/SupplementaryTableS1.xlsx. (XLSX 86 kb)

## Abbreviations

AUC: Area under the curve
CP: Cyclic peptide drug dataset
ELS: Enumerating lasso solutions
FBS: Forward beam search
HSA: Human serum albumin
LOOCV: Leave-one-out cross-validation
MAE: Mean absolute error
PCA: Principal component analysis
PEOE: Partial equalization of orbital electronegativity
PISA: π (carbon and attached hydrogen) component of the solvent accessible surface area
PPB: Plasma protein binding
RMSE: Root mean squared error
SCP: Synthetic cyclic peptide dataset
SM: Small molecule drug dataset
